# The Development of C9orf72-Related Amyotrophic Lateral Sclerosis and Frontotemporal Dementia Disorders

**DOI:** 10.3389/fgene.2020.562758

**Published:** 2020-09-02

**Authors:** Qijie Yang, Bin Jiao, Lu Shen

**Affiliations:** ^1^Department of Neurology, Xiangya Hospital, Central South University, Changsha, China; ^2^National Clinical Research Center for Geriatric Disorders, Central South University, Changsha, China

**Keywords:** C9orf72, ALS, FTD, development, treatment

## Abstract

The expanded GGGGCC hexanucleotide repeat in the non-coding region of the *C9orf72* gene is the most common genetic cause of amyotrophic lateral sclerosis (ALS) and frontotemporal dementia (FTD). There are three main disease mechanisms: loss of function of C9ORF72 protein, gain of function from the accumulation of sense and antisense (GGGGCC)n in RNA, and from the production of toxic dipeptides repeat proteins (DPRs) by non-AUG initiated translation. While many of the downstream mechanisms have been identified, the specific pathogenic pathway is still unclear. In this article, we provide an overview on the currently available literature and propose several hypotheses: (1) The pathogenesis of *C9orf72*-associated ALS/FTD, which cannot be explained by a single mechanism, involves a dual mechanism of both loss and gain of function. (2) The loss of function and gain of function can cause TDP-43 aggregation and damage nucleocytoplasmic transport. (3) Neurodegeneration can be caused by an accumulation of toxic substances in neurons themselves. In addition, we suggest that microglia may cause neurodegeneration by releasing inflammatory factors to neurons. Finally, we summarize several of the most promising treatment strategies.

## Introduction

Amyotrophic lateral sclerosis (ALS) is a chronic progressive and fatal neurodegenerative disease caused by the degeneration of upper and lower motor neurons. It is characterized by progressive muscle weakness and atrophy, eventually leading to respiratory failure. Approximately 5–50% of ALS patients have clinical symptoms of frontotemporal dementia (FTD) (Hudson, 1981; Lomen-Hoerth et al., 2003). FTD is the second most common form of early-onset dementia, manifesting as frontal and/or temporal lobe atrophy, accompanied by personality and behavioral changes as well as language dysfunction. In fact, a proportion of patients with FTD also develop ALS. In addition to clinical overlapping, ubiquitin-positive tau-negative inclusion bodies (TDP-43), were considered to be a major pathological protein in ALS and FTD pathological studies (Neumann et al., 2006). In 2011, a major discovery connecting ALS and FTD was made that the expanded GGGGCC hexanucleotide repeat of the *C9orf72* gene is an important genetic cause for ALS/FTD, accounting for roughly 40% of familial ALS patients, 25% of familial FTD patients and as high as 88% in familial ALS/FTD patients (DeJesus-Hernandez et al., 2011). ALS and FTD present significant clinical, genetic, and histopathological overlaps; therefore, they are considered as two extremes of the same disease continuum.

**FIGURE 1:**
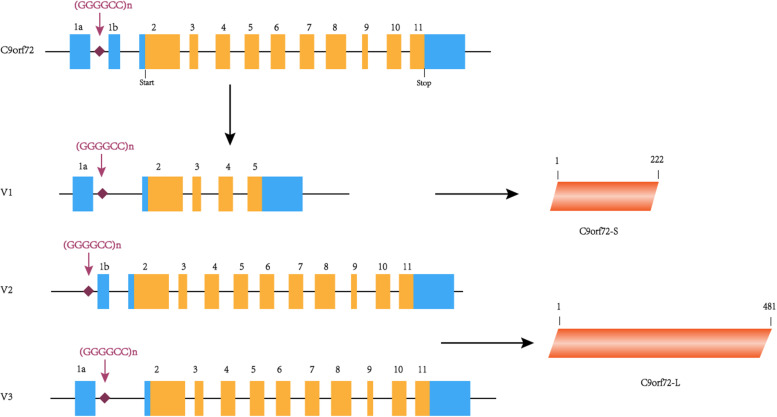
C9orf72 gene, transcript variants and protein isoforms. In this figure, coding exons are shown in orange, and non-coding exons are shown in blue. The C9orf72 gene contains 11 exons and (GGGGCC)n is located between exons 1a and 1b. (GGGGCC)n is located in the first intron of V1 and V3 and in the promoter region of variant 2. C9orf72-S is 222 amino acids protein encoded by V1. C9orf72-L is 481 amino acids protein encoded by V2 and V3.

The *C9orf72* gene (chromosome 9 open reading frame 72 gene) is located on the short arm of chromosome 9 and is transcribed into three major transcripts (V1, V2, and V3), which produce the two protein isoforms [C9ORF72-S (C9-S) and C9ORF72-L (C9-L)]. The GGGGCC hexanucleotide repeat mutation is located on intron 1 between exons 1a and 1b (Figure 1). In a healthy individual, the GGGGCC expansion can range from 2–30 repeats, but in patients with *C9orf72*-associated ALS/FTD, it ranges from several hundred to thousands of repeats (DeJesus-Hernandez et al., 2011). Since expansions of 20 to several hundred units are found in both healthy and diseased individuals, the minimum number for pathological GGGGCC expansions is still unclear (Gami et al., 2015). Interestingly, GGGGCC pathological repeat expansions are rarely observed in the Han Chinese population, with frequencies of 0–4.8% in ALS cases, whereas the highest frequencies are observed in European populations (Cruts et al., 2013). Unfortunately, there is currently no effective treatment for this disease, highlighting the need for more research exploring the potential mechanism of *C9orf72*-mediated ALS/FTD in order to find therapeutic targets. Therefore, this article summarizes the previous literature focused on existing research on the pathogenesis, which will help to explore effective treatment methods.

### C9orf72-Specific Mechanisms

**FIGURE 2:**
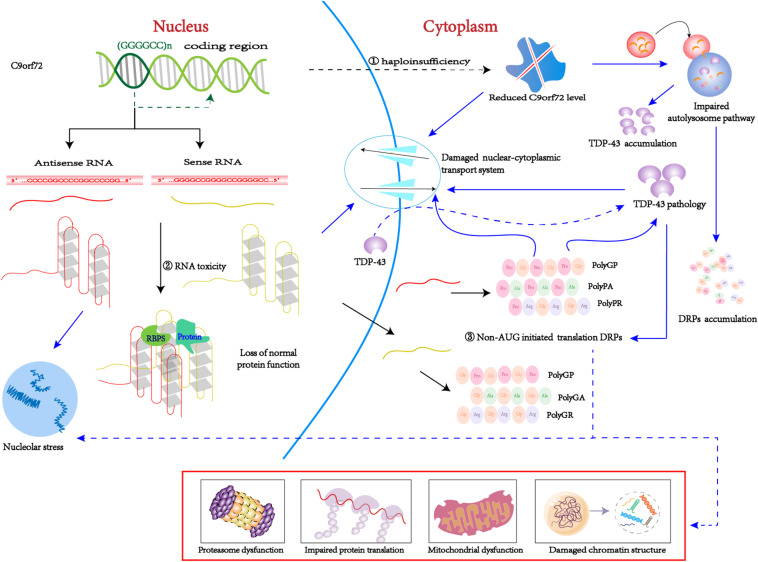
C9orf72 pathological pathways. ➀ Due to epigenetic modification, C9orf72 protein haploinsufficiency and loss of function. ➁ The (GGGGCC)n RNA with a G-quadruplexes structure can isolated the RNA bind proteins, result in the loss of normal function. ➂ Through non-AUG-initiated translation, antisense and sense RNA will produce toxic DPR. Purple arrow indicates possible downstream mechanism; As mentioned above, C9-L and C9-S are related to the autophagy-lysosome pathway and the nucleocytoplasmic transport, respectively. On the one hand, reduced C9orf72 will damage nucleocytoplasmic transport, which will promote TDP-43 migrates from the nucleus to the cytoplasm. TDP pathology and DRPs form a positive feedback loop. On the other hand, reduced C9orf72 impairs the autolysosome pathway, thereby clearing DRPs and TDP-43 dysfunction and causing accumulation in the body. In addition, (GGGGCC)_n_ RNA can bind to important proteins in biological processes, such as RanGAP1 and nucleolin, thus disrupting nucleocytoplasmic transport and nucleolus stress. DRPs have many possible toxic pathways. Poly (GR) can impair protein translation by interacting with ribosomal subunits and inducing the formation of SGs. Poly (GR) can damage mitochondrial function by promoting the degradation of Atp5a1 and Poly (PR) adversely influences heterochromatin structure, inducing repetitive element expression and the accumulation of double-stranded RNA. Poly(GA) impair proteasome function by recruitting 26S proteasomes.

The pathogenesis of *C9orf72*-mediated ALS/FTD is associated with both a loss and gain of function, including the following: (1) loss-of-function mechanism: decreased transcription of the *C9orf72* coding region that leads to reduced production of the C9ORF72 protein, also known as C9ORF72 haploinsufficiency; (2) gain-of-function mechanism: sense and antisense (GGGGCC)_n_ RNA can sequester or alter the function of RNA-binding proteins and other proteins; (3) By non-AUG initiated translation: sense and antisense (GGGGCC)_n_ RNA produce five toxic dipeptides repeat proteins (DPRs) (Figure 2). Based on the particular combination of these mechanisms, they can have more or less influence on the disease.

#### Loss-of-Function Mechanisms

In a large-scale *C9orf72* expansion carrier study, V1 and V2 (especially V2) in the frontal cortex and cerebellum were reduced compared to the control group, with no reduction in V3 observed (van Blitterswijk et al., 2015). Further, the loss of *C9orf72* orthologs in *Caenorhabditis elegans* as well as zebrafish has been shown to cause degeneration of motor neurons (Ciura et al., 2013; Therrien et al., 2013). Together, this supports the hypothesis that haploinsufficiency of the C9ORF72 protein may underlie *C9orf72*-mediated ALS/FTD. Epigenetic modifications may also contribute to the reduction of C9ORF72 protein, such as CpG island hypermethylation in the promoter region of *C9orf72*, or methylation of the *C9orf72* repeat expansion itself, or possibly even histone trimethylation (Belzil et al., 2013, 2014; Xi et al., 2013, 2015).

However, the consequences of reduced C9ORF72 protein level are not well understood. In fact, very little is known about C9ORF72 protein and its function. At the molecular level, the major predicted structural feature of the C9ORF72 protein is a DENN domain which is a GDP/GTP exchange factor (GEF) that activates Rab-GTPases (Levine et al., 2013). Further research has found that the C9-L isoform of the C9ORF72 protein contains the longin-DENN-Alpha domains, whereas the C9-S isoform only contains the longin domain (Xiao et al., 2016). Only the C9-L isoform can interact with another protein containing the DENN domain such as SMCR8 or WD41(WD40 repeat protein) to form a tight protein complex, which plays an important role in regulating the autophagy-lysosome pathway (Amick et al., 2016; Sullivan et al., 2016; Xiao et al., 2016). It is well known that p62 is an autophagy protein, also called SQSTM1 protein. Importantly, ubiquitin and p62-positive inclusion bodies are found in ALS / FTD cases, further supporting the idea that the autophagosome-lysosome system is impaired. C9-S is located on the nuclear membrane of healthy neurons and may interact directly with Importin−β1 or Ran−GTPase (Xiao et al., 2015). Therefore, it is predicted that C9-S may act as a nuclear transport protein. Combined, this suggests that the C9-L isoform is involved in the autophagy-lysosome pathway, while C9-S is involved in nucleocytoplasmic transport.

**FIGURE 3:**
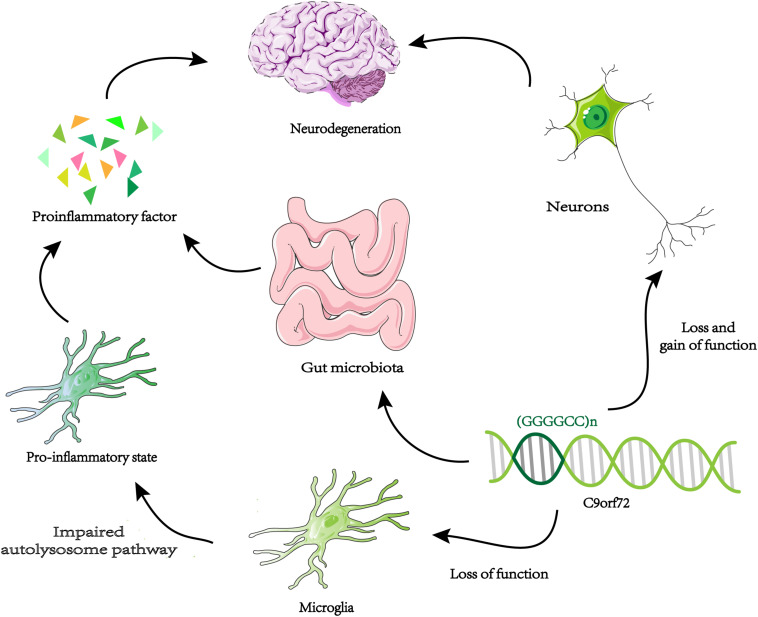
Neurodegeneration and microglia inflammation. Decreased C9orf72 expression in microglia, resulting in autolysosome pathway dysfunction, and release proinflammatory factor. By the loss and gain of function, toxic substances (DRPs, TDP-43, etc.) accumulate in neurons. The two approaches may be uniquely combined to promote neurodegeneration in ALS / FTD. When the expression of C9orf72 decreases, intestinal microorganisms will cause pathological inflammation of the brain.

A new way to study gene function is to identify genetic interaction pairs through synthetic lethal screening. Synthetic lethality means that mutants carrying two specific genes cannot survive and when they mutate separately will not cause fatal damage. So these genetic interaction pairs usually have similar characteristics and functions in single pathways, or work in parallel pathways. Through genome-wide synthetic lethal CRISPR screen in *C9orf72* knockout cells, *FIS1* and *C9orf72* were identified as a synthetic lethal interaction (Chai et al., 2020). Without C9ORF72, FIS1 can repress the secretion of inflammatory cytokines in parallel pathways (Chai et al., 2020). In summary, C9ORF72 may have anti-inflammatory effects. Knocking down *C9orf72* expression in mice produces an upregulation of microglial activation genes (Lagier-Tourenne et al., 2013). Microglia from *C9orf72*^–^/^–^ mice displayed endosomal-lysosomal dysfunction and were found to have elevated levels of cytokines IL-6 and IL-1b, leading to a pro-inflammatory state (O’Rourke et al., 2016). Therefore, decreased expression of *C9orf72* in microglia can directly change its function and lead to inflammation of the nervous system. It is possible that the reduction of *C9orf72* expression in microglia produces an abnormal level of inflammation of the central nervous system, which coincides with increased toxicity in neurons leading to neurodegeneration (Figure 3). The above results all prove that the function of *C9orf72* is closely related to inflammation. Surprisingly, the researchers found that *C9orf72*^–^/^–^ mice at the Harvard Institute exhibited autoimmune and inflammatory phenotypes, while *C9orf72*^–^/^–^ mice at Broad Institute had significantly reduced inflammation phenotypes (Burberry et al., 2020). The results indicated that when *C9orf72* function declines, the environment may be an important regulator of the inflammatory phenotype. By using broad-spectrum antibiotics to reduce the microbial burden of mutant mice and transplanting intestinal flora from a protective environment will reduce the inflammatory phenotype of *C9orf72*^–^/^–^ mice. A novel opinion was proposed that the function of C9ORF72 is to prevent pathological inflammatory reaction caused by microbial community (Burberry et al., 2020; Figure 3). We need to make more efforts to explore the link between genetic and environmental factors in ALS.

Mice share a 91.89% nucleotide and a 98.75% amino acid level similarity with the human *C9orf72* ortholog coding regions (Xiao et al., 2016). In a series of loss-of-function mouse models, *C9orf72* knockout had little association with neurodegeneration, and instead had a strong relationship with lymphadenopathy and splenomegaly (Burberry et al., 2016; Jiang et al., 2016; O’Rourke et al., 2016; Sudria-Lopez et al., 2016). Importantly, none of the mouse *C9orf72* knockouts displayed an ALS or FTD phenotype, indicating that the deletion of *C9orf72* alone is not sufficient to induce these disorders. But gain-of-function disease processes may trigger neurodegeneration through mechanisms induced by reduced C9ORF72 levels. In ALS/FTD Human Induced Motor Neurons (iMNs), *C9orf72* haploinsufficiency may increase glutamate receptors on the surface of iMNs, leading to excitotoxicity and impaired clearance of DPRs, leading to neurotoxicity, ultimately resulting in neurodegeneration (Shi et al., 2018). The question of whether there is a synergy between loss-of-function and gain-of-function mechanisms that mediate the pathogenesis of c9-FTD/ALS has aroused intense debate in this field. Zhu et al., establish mouse carrying a bacterial artificial chromosome (BAC) containing a human C9orf72 gene with a repeat expansion, which cannot produce the human C9ORF72 protein. Then, a *C9orf72* dual gain-and-loss mouse model is established by mating C9^450C^ mice to mice with inactivation of one or both endogenous C9orf72 alleles. This new mouse model has an endogenous mouse C9ORF72 protein expression level and repeat expansion (C9^450C^ *C9orf72*+/−,C9^450C^ *C9orf72*^–^/^–^). Compared with C9^450C^ mice, C9^450C^ *C9orf72*+/− and C9^450C^ *C9orf72*^–^/^–^mouse showed more severe learning, memory and motor deficits (Zhu et al., 2020). In hippocampal CA1, the poly (GP) and poly (GA) are dose-dependent with C9ORF72, with higher levels accumulating in C9^450C^ *C9orf72*^–^/^–^mice (Zhu et al., 2020). Thus, Zhu et al., proposed that C9ORF72 protein haploinsufficiency exacerbates a gain of toxicity from repeat expansion of C9orf72 in ALS/FTD. Very recently, Boivin et al. (2020) have also found that reduced C9ORF72 levels leads to an accelerated accumulation of DPRs. It is possible that neurons clear DPRs through a select autophagy pathway and that a reduction in C9ORF72 levels in neurons impairs autophagy, resulting in the accumulation of DPRs, eventually leading to neurodegeneration.

#### Gain-of-Function Mechanisms

Pathological studies in ALS/FTD have found that sense and antisense RNA foci as well as p62-positive and TDP-43-negative inclusions (DPRs), are widely distributed throughout the CNS, supporting the involvement of gain-of-function mechanisms (DeJesus-Hernandez et al., 2011; Gendron et al., 2013; Zu et al., 2013). Additionally, research has found that RNA foci and DPRs involve a wide range of downstream mechanisms, but no clear pathway has emerged.

##### RNA toxicity-mediated function acquisition mechanism

The first reported RNA foci were in Myotonic dystrophy type 1 (DM1) patients, which is caused by a CTG repeat expansion of the *DMPK* gene and is a typical RNA toxicity disease. DM1 is believed to be caused by RNA transcripts (RNA foci) isolating crucial RNA-binding proteins (MBNL1, CUGBP1, etc.), leading to cause widespread splicing abnormalities (Gomes-Pereira et al., 2011).

Just as in DM1, RNA foci have also been observed in *C9orf72*-associated FTD/ALS patients thus promoting research into possible RNA toxicity mechanisms. RNA foci are formed by the transcription of (GGGGCC)_n_ repeats in the sense or antisense direction. Sense RNA foci exist in most brain regions, such as cortical regions, granule cells of the hippocampal dentate gyrus, and cerebellum. However, antisense RNA foci appear to be most common in cerebellar Purkinje cells and spinal motor neurons (Cooper-Knock et al., 2015; DeJesus-Hernandez et al., 2017). RNA foci often coincide in the areas affected by ALS / FTD. Moreover, RNA toxicity was revealed in zebrafish model for C9orf72 ALS. Injecting sense (GGGGCC)_n_ and antisense (CCCCGG)_n_ into zebrafish embryos can independently induce a motor axonopathy in the absence of DPRs (Swinnen et al., 2018). From ∼ 35 repeats on, axonal outgrowth was reduced and axonal branching was abnormal and the antisense repeat RNA was less toxic than the sense repeats (Swinnen et al., 2018). Several mechanisms underlying RNA toxicity are possible. *In vitro* and *in vivo*, the (GGGGCC)_n_ repeats can form both G-quadruplexes (G-Qs) and hairpins (Haeusler et al., 2014; Zhou et al., 2018). These RNA structures can sequester proteins essential for normal RNA processing, resulting in extensive abnormal RNA metabolism. Evidences have shown that RNA foci co-localize with a wide range of RNA-binding proteins, like nucleolin, Pur-α, hnRNPs, ADARB2, SRSF1, SRSF2, and ALYREF (Donnelly et al., 2013; Xu et al., 2013; Cooper-Knock et al., 2014; Haeusler et al., 2014; Stepto et al., 2014). The (GGGGCC)_n_ repeats might induce possible nucleolar stress, by sequestration of nucleolin which is an essential nucleolar protein (Haeusler et al., 2014). In addition, (GGGGCC)_n_ RNA can impair nucleocytoplasmic transport by binding with RanGAP1, a key regulator of nucleocytoplasmic transport (Zhang et al., 2015). Moreover, RNA binding protein (RBP) plays multiple roles in splicing, translation regulation, RNA transport, and degradation. Therefore, the (GGGGCC)_n_ RNA may impair numerous cellular processes including nucleocytoplasmic transport, as well as induce nucleolar stress and disrupt RNA splicing.

Nevertheless, studies have questioned the mechanism of RNA toxicity. Pure repeats can generate DPRs caused eye degeneration (Mizielinska et al., 2014). Regularly interspersed stop codons are introduced into Drosophila to form RNA-only repeats which has RNA G-quadruplexes and cannot translate dipeptide repeat proteins. It was found that similar effects could not be produced under the same conditions (Mizielinska et al., 2014). Consistent with this, using novel RNA-only Drosophila models, researchers have found that cytoplasmic and nuclear sense or antisense RNA are not toxic even though these RNA foci can isolate endogenous Drosophila RNA-binding proteins (Moens et al., 2018). Therefore, further studies are needed to explore the mechanisms of RNA toxicity in more detail.

##### Repeated protein-mediated function acquisition mechanism

Human neuropathological studies have observed p62-positive and TDP-43-negative inclusions in *C9orf72*-associated ALS/FTD families (Boxer et al., 2011). After these pathogenic features were identified, researchers demonstrated the inclusions were byproducts of repeat-associated non-AUG (RAN) translation (Josephs et al., 2011; Ash et al., 2013). The sense or antisense (GGGGCC)_n_ RNAs is translated by RAN-initiated translation to produce toxic DPRs, poly-GA, poly-GP, poly-GR from the sense chain, poly-PA, poly-GP, and poly-PR from the antisense chain (Josephs et al., 2011; Mann et al., 2013; Mori et al., 2013).

In a recent study, Mizielinska et al. (2014) generated “protein-only” constructs that use the alternative codon found in G4C2 repeats to generate poly (GR), poly (PR), poly (GA), or poly (PA) and then expressed them in the eyes of flies. The constructs containing 36 dipeptide repeats produced the arginine-containing poly(GR)_36_ and poly(PR)_36_ causing eye degeneration and death, while poly(GA)_36_ and poly(PA)_36_ had no effect (Mizielinska et al., 2014). It shows that DPRs containing arginine poly (GR), poly (PR) are toxic. At the same time, in adult neurons, researchers using longer protein-only sequences compared with 36 dipeptide repeats found that poly (PR)_100_ and poly(GR)_100_ cause a significant decrease in survival rate (Mizielinska et al., 2014). In a mammalian model, (GR)_50_ mice and (PR)_100_ mice have exhibited neurodegeneration and behavioral deficits, which gradually aggravated over time, suggesting age-dependent neurodegeneration (Zhang Y. J. et al., 2018; Zhang et al., 2019). Nearly 60% of the mice died prematurely, and the others suffered memory and behavior impairment after (PR)_50_ was administered to the ventricle via AVV (Zhang et al., 2019). Actually, premature death mice have higher PR levels than surviving mice. Thus, arginine-containing DPRs are toxic and the increase in expression will aggravate toxicity.

However, the cellular pathway(s) that arginine-containing DPRs function through are largely unknown. Through proteomic analysis, poly(GR)_50_ and poly(PR)_50_ have been found to interact with RBP’s as well as proteins with low complexity sequence domains (LCD), thus damaging the assembly, dynamics, and functions of membraneless organelles (such as nucleoli, stress particles, and nuclear pore complexes) (Lee et al., 2016). Accordingly, it has been shown that arginine-rich DPRs disrupt nucleocytoplasmic transport, cause nucleolar stress, and induce assembly of stress granules (SGs) (Freibaum et al., 2015; Boeynaems et al., 2017; Mizielinska et al., 2017). Under stress conditions, cells inhibit mRNA translation, forming membraneless SGs in cytoplasm, which are aggregated by a large number of proteins and RNA. Further research has found that many nucleocytoplasmic transport factors are recruited to SGs, and that SGs can disrupt nuclear and cytoplasmic transport (Zhang K. et al., 2018). Through establishing a mouse model of C9ORF72-related ALS/FTD, we can further understand the exact pathogenic effects of arginine-containing DPRs. Poly (GR) can impair protein translation in mouse models through two ways: interacting with ribosomal subunits and inducing the formation of SGs (Zhang Y. J. et al., 2018). Poly (GR) can increase the degradation of Atp5a1 via the proteasome pathway and decreases Atp5a1 levels which compromised mitochondrial morphology and function (Choi et al., 2019). Poly (PR) can disrupted HP1α liquid phases and cause abnormal histone methylation, which adversely influences heterochromatin structure (Zhang et al., 2019). Heterochromatin is a bona fide factor of silent chromatin and HP1α is required for establishing and maintaining the structure of heterochromatin. Consequently, poly (PR) induced repetitive element expression and the accumulation of double-stranded RNA, contributing to the neurodegeneration (Zhang et al., 2019).

In addition to the toxicity of arginine-containing DPRs, poly (GA) also show toxicity in heterologous cells, primary neuronal cultures and mice. Poly(GA) aggregates form twisted ribbons *in situ* neuronal which can recruit 26S proteasomes and impair proteasome function, consequently abnormal protein homeostasis (Guo et al., 2018).

However, strong objections have been put forward that DPRs play a major and exclusive pathological role in *C9orf72*-associated ALS/FTD (Mackenzie et al., 2015). Although DPR inclusions are widely distributed in the brain regions of *C9orf72*-associated ALS/FTD patients, few inclusions are found in the spinal cord in *C9orf72*-associated ALS from post-mortem studies, challenging their contribution to *C9orf72*-associated ALS cases (Gomez-Deza et al., 2015). Nevertheless, DPRs may still play a role in the early stage of the disease, considering that autopsy studies may not reflect the early pathogenicity of DPRs.

### C9orf72-Associated Mechanism: TDP-43, Nucleocytoplasmic Transport

RNA foci, DPR inclusions, and TDP-43 aggregation are three main features of *C9orf72*-associated ALS/FTD neuropathology. TDP-43 is a widely expressed nuclear RNA-binding protein that participates in a variety of cell functions. In healthy cells, TDP-43 mostly resides in the nucleus, while in *C9orf72*-associated ALS/FTD patients, almost all neurons with cytoplasmic TDP-43 inclusions have an accompanied depletion of nuclear TDP-43. Human neuropathological studies reveal that TDP-43 pathology is correlated with clinical phenotype and the degeneration of key anatomical regions (Mackenzie et al., 2013; Davidson et al., 2016). Rare cases provide unique insights for understanding the role of TDP-43 in *C9orf72*-associated ALS/FTD. For example, patients carrying a *C9orf72* 30-unit repeat expansion have intracranial DPR inclusions and RNA foci, but no TDP-43 inclusions or clinical symptoms (Gami et al., 2015). Comparing archival surgical resection tissue from a patient who underwent left anterior temporal lobectomy 5 years prior to the onset of FTD symptoms with that of tissue from the autopsy, it was found that DPR inclusions and RNA foci were present in the surgical specimens, but almost no TDP-43 inclusions, whereas the patient had large amounts of TDP-43 inclusions postmortem (Vatsavayai et al., 2016). Additionally, in a mouse model carrying a *C9orf72* hexanucleotide amplification where RNA foci and DPRs cannot drive degeneration, but in the presence of TDP-43 pathology, neuronal loss and abnormal behavior occurred (Chew et al., 2015; Peters et al., 2015). Taken together, this indicates that RNA foci and DPRs may only be present at the early stages of *C9orf72*-associated ALS/FTD, while TDP-43 pathology occurs later leading to neuronal degeneration. However, the question of whether there is a relationship between C9ORF72 protein reduction, RNA foci, DPRs, and TDP-43 pathology remains.

Proteins enter and exit the nucleus through the nuclear pore complex (NPC), and are regulated by nuclear transport receptors knowns as transport factors (TFs), most of which are in the karyopherin family, such as importins that facilitate entry into the nucleus and exportins that facilitate exit from the nucleus. In addition, this process involves the Ras-related nuclear protein (Ran)GTP-RanGDP cycle, in which the small GTPase Ran regulates cargo across the nuclear membrane through its own nucleotide state, and RanGAPs, RanBPs, and RCC1 play an important role in the nucleotide transformation of Ran (Kim and Taylor, 2017). Therefore, the formation of TDP-43 pathology requires the transport of TDP-43 from the nucleus to the cytoplasm. For example, studies in human tissues have shown that the loss of C9−S, Importin−β1, and Ran−GTPase from the nuclear membrane is associated with nuclear depletion and cytoplasmic mislocalization of TDP−43 in neurons (Xiao et al., 2015). As mentioned earlier, RanGAP1 can bind to the (GGGGCC)_n_ RNA, thus damaging nucleocytoplasmic transport (Zhang et al., 2015).

Arginine-containing DPRs may target membraneless organelles such as the NPC, which in turn affects nucleocytoplasmic transport. Jovièiæ et al. (2015) identified a striking number of modifier genes involved in nucleocytoplasmic transport from two unbiased genome wide screens in yeast for suppressors and enhancers of PR toxicity. Freibaum et al. (2015) also found 18 genetic modifiers in transgenic fly lines. These modifiers include the NPC components, proteins that coordinates the export of nuclear RNA and the import of nuclear proteins, enzymes involved in (Ran)GTP-RanGDP cycle. Additionally, these modifier genes involved in nuclear transport has been proven could also modify C9orf72 DPR toxicity *in vivo* (Boeynaems et al., 2016). After knock downing the Trn encoding transportin 1 in flies expressing PR, the neuronal RNA-binding protein, Elav, further increased cytoplasmic localization and decreased nuclear staining (Boeynaems et al., 2016). In the cortex of (GA)_50_ mice, poly (GA) can sequester nucleocytoplasmic transport proteins into inclusion, such as RanGAP1 and Pom121 (Zhang et al., 2016). In Drosophila has found that G4C2-derived DPRs causes the cytoplasmic mislocalization of TDP-43. Timing and extent of TDP-43 dysfunction depends on the number and characteristics of dipeptide-repeat proteins rather than repeat length, whereas poly-GR causes early onset and poly-GA / poly-GP causes late onset (Solomon et al., 2018). All the above-mentioned supports the idea that C9ORF72 protein reduction, DPRs, and RNA foci are related to TDP-43 pathology, either directly or indirectly.

Recently it has been found that cytoplasmic accumulation of TDP-43 can directly trigger nucleocytoplasmic transport defects by disrupting the localization of nucleoporins (Nups) and TFs (Chou et al., 2018). The NPC is composed of more than 30 Nups and can regulate the receptor-mediated nucleocytoplasmic transport of macromolecules. Importantly, cytoplasmic accumulation of soluble TDP-43 results in karyopherin-α2/4 (KPNA2/4) pathology and an increase in DPRs levels (Solomon et al., 2018). KPNA2 and KPNA4 are members of the karyopherin-α family and are part of this classical nuclear import pathway.

The loss of C9-S, formation of RNA foci, or the accumulation of DPRs might promote cytoplasmic mislocalization of TDP-43. Cytoplasmic accumulation of TDP-43 exacerbates nucleocytoplasmic transport defects in a positive feedback loop and at the same time enhances levels of DPRs, thus leading to a vicious circle (Figure 2). Therefore, TDP-43 pathology and nucleocytoplasmic transport seem to be a key point along the common path of the mechanism underlying the loss and gain of function.

### Therapeutic Strategies

To date, *C9orf72*-associated ALS/FTD has no effective treatment. The only two pharmacological treatments approved by the USA Food and Drug Administration for ALS treatment are riluzole and edaravone. Here, we introduce several of the most promising novel treatment strategies below.

#### Gene Silencing Strategy

Gene silencing is a good way to deal with *C9orf72*-associated toxicity acquisition mechanisms, such as the use of antisense oligonucleotides (ASO) or RNA interference (RNAi). ASO is a short DNA/RNA sequence complementary to the target gene mRNA. Through the RNase H-dependent pathway, an ASO combines with a complementary target mRNA, which activates RNaseH leading to mRNA degradation and thus inhibiting the production of a given protein. Alternatively, through steric blocking it can prevent RNA splicing/processing without degradation. Both mechanisms can reduce protein expression of target genes. However, the pathogenesis of *C9orf72*-associated ALS/FTD, which cannot be explained by a single mechanism, involves a dual mechanism of both loss and gain of function. Therefore, an important goal of ASOs targeting C9orf72 is to reduce the expression of (GGGGCC)_n_ mutations while maintaining the expression of normal alleles. Some studies have shown that ASOs can target (GGGGCC)_n_ without lowering the mRNA level of C9orf72 mRNA, significantly reducing the number of RNA foci and the percentage of cells containing RNA foci in each cell in induced pluripotent stem cells (iPSCs) from C9orf72 ALS patients (Donnelly et al., 2013). At the same time, a single injection of targeted (GGGGCC)_n_ ASOs into the ventricle of C9^450*B*^ transgenic mice can continuously reduce RNA foci and DPRs in the cortex and spinal cord, and a single dose of ASO treatment can improve the behavioral defects of C9^450*B*^ transgenic mice (Jiang et al., 2016). Although the role of RNA foci or DPRs in neurodegeneration is uncertain, ASO therapy can reduce both potential toxicities. According to the above research, Biogen and Ionis Company develop the ASO BIIB078 which is currently undergoing Phase 1 clinical trial by intrathecal injection (clinicaltrials.gov; NCT03626012). Another method of gene silencing is through the use of an RNAi strategy. RNAi works when a double-stranded DNA homologous to an endogenous mRNA is introduced into the cell, the mRNA is degraded. RNAi will be described below in gene therapy.

#### Small Molecule Compound Treatment Strategies

Some small molecule compounds can act on a specific pathway and rescue part of the toxicity seen with *C9orf72*-associated ALS/FTD. For example, TMPyP4 and KPT-276 can restore nucleocytoplasmic trafficking. Tauroursodeoxycholic acid (TUDCA) and salubrinal can decrease ER stress (Mis et al., 2017). Additionally, small aromatic compounds can target RNA G-quadruplexes and reduce RNA foci (Schludi and Edbauer, 2018). Arginine-rich dipeptide repeats can inhibit nonsense-mediated mRNA decay (NMD) and tranilast can suppress dipeptide repeat toxicity by activating NMD pathway (Xu et al., 2019). However, as mentioned above, the pathogenesis of *C9orf72*-associated ALS/FTD involves multiple pathways, and these small molecule compounds only play a role in a single pathway, thereby limiting its therapeutic effects. Recently, scientists have discovered that the hairpin structure is the main contributor to RNA translation in *in vitro* studies and the G-quadruplex ligands cannot inhibit RNA translation, and proposed that the hairpin structure instead of G-quadruplex will become a new treatment strategy (Wang et al., 2019). Compound 4 can selectively bind to the hairpin form and can block polysome assembly, and inhibiting the formation of RNA-protein complexes thereby reducing DPR proteins and RNA foci.

#### Gene Therapy

Gene therapy has made breakthroughs in many diseases, and is considered among our best hope for the cure of genetic-based diseases. For diseases characterized by a gain of function, genes encoding regulatory peptides or nucleotides with gene silencing function, like RNAi and ASOs, can be delivered. For diseases characterized by loss of function, wild-type genes can be delivered to restore normal functioning.

Gene therapy is usually combined with gene silencing in order to target toxic gain-of-function diseases. The key point of gene therapy is to use effective but non-invasive strategies to deliver regulatory peptides or nucleotides to the central nervous system. In order to achieve therapeutic effects in patients with ALS and FTD, extensive transduction of neurons (including glial cells) in the brain and spinal cord is required. Adeno-associated viruses (AAVs) are a promising method of gene therapy for neurological diseases. Different serotypes of AAVs have different tissue tropism and transduction efficiencies. For example, after intravenous administration, AAV9 has the ability to cross the blood–brain barrier and tends to transduce glial cells while AVV6 preferentially transduces neurons (San Sebastian et al., 2013; Borel et al., 2014; Dirren et al., 2015). Evidences showed that AAV5 can transduce different types of neuronal cells and AAV5-delivered artificial RNAi can reduce the accumulation of repeat-containing *C9orf72* transcripts (Martier et al., 2019). However, non-allele-specific RNAi strategies may lead to excessive silencing of normal alleles. Alternatively, gene therapy may represent a great strategy to rescue *C9orf72* haploinsufficiency. It must be pointed out that a loss-of-function mechanism alone cannot account for the disease’s pathogenesis. Therefore, in this case, an alternative treatment strategy is necessary, such as using a non-allele-specific RNAi to silence both normal and mutant alleles, while simultaneously replacing normal genes.

Recently, Sangamo Therapeutics designed allele-specific zinc finger protein transcription factors (ZFP-TFs) to target the pathogenic CAG repeat in Huntington’s disease (Zeitler et al., 2019). Once a ZFP-TF is combined with the target sequence of DNA, it can suppress the expression of that gene. Moreover, it shows allele-selective repression of the *HTT* gene, as it has high specificity for CAG repeats of mutant HTT genes but no effect on normal genes. Excitingly, two companies, Sangamo Therapeutics and Pfizer, are currently collaborating on the use of ZFP-TF technology to treat *C9orf72*C9-associated ALS/FTD.

## Conclusion

In view of the important position of *C9orf72* repeat expansion in ALS and FTD, uncovering the mystery of *C9orf72*C9-associated ALS/FTD will help us find effective therapeutic targets, thereby having a significant influence on patients suffering from these debilitating neurodegenerative diseases.

## Author Contributions

QY wrote the manuscript. BJ and LS made suggestions and edited the manuscript. All authors read and approved the final manuscript.

## Conflict of Interest

The authors declare that the research was conducted in the absence of any commercial or financial relationships that could be construed as a potential conflict of interest.
